# Supplementary material for: Mechanisms governing the pioneering and redistribution capabilities of the non-classical pioneer PU.1
Source: Nat Commun. 2020 Jan 21;11:402. doi: 10.1038/s41467-019-13960-2 (PMC6972792; doi:10.1038/s41467-019-13960-2)
Supplement: Supplementary file 7 — Source data [file 41467_2019_13960_MOESM7_ESM.zip › Source_Data/FigureS2/FigureS2B_MotifScanOutput/KmeansCluster4/homerResults/motif14.similar.html]

motif14

## Information for motif14

A
C
G
T
A
T
C
G
A
G
T
C
A
G
C
T
A
C
T
G
A
C
G
T
A
G
C
T
  
Reverse Opposite:  

C
T
G
A
G
T
C
A
A
G
T
C
C
T
G
A
A
C
T
G
A
T
G
C
C
G
T
A
  

|  |  |
| --- | --- |
| p-value: | 1e-59 |
| log p-value: | -1.375e+02 |
| Information Content per bp: | 1.881 |
| Number of Target Sequences with motif | 1125.0 |
| Percentage of Target Sequences with motif | 24.92% |
| Number of Background Sequences with motif | 6265.3 |
| Percentage of Background Sequences with motif | 14.99% |
| Average Position of motif in Targets | 104.8 +/- 55.9bp |
| Average Position of motif in Background | 99.4 +/- 70.6bp |
| Strand Bias (log2 ratio + to - strand density) | 0.0 |
| Multiplicity (# of sites on avg that occur together) | 1.08 |
| Motif File: | file (matrix) reverse opposite |

### Similar de novo motifs found

|  |  |  |  |  |  |  |  |
| --- | --- | --- | --- | --- | --- | --- | --- |
| Rank | Match Score | Redundant Motif | P-value | log P-value | % of Targets | % of Background | Motif file |
| 1 | 0.773 | C T A G C T G A A C T G C G T A C G T A A G T C C G T A A C T G A G T C | 1e-25 | -58.710871 | 3.72% | 1.35% | motif file (matrix) |
| 2 | 0.807 | A C G T A C T G A G T C A T G C A C T G A C G T A C G T G A C T | 1e-22 | -50.982172 | 8.26% | 4.65% | motif file (matrix) |
